# New Coleoptera records for New Brunswick, Canada: Kateretidae, Nitidulidae, Cerylonidae, Endomychidae, Coccinellidae, and Latridiidae

**DOI:** 10.3897/zookeys.179.2581

**Published:** 2012-04-04

**Authors:** Reginald P. Webster, Jon D. Sweeney, Ian DeMerchant

**Affiliations:** 1 Natural Resources Canada, Canadian Forest Service - Atlantic Forestry Centre, 1350 Regent St., P.O. Box 4000, Fredericton, NB, Canada E3B 5P7

**Keywords:** Cerylonidae, Coccinellidae, Endomychidae, Kateretidae, Latridiidae, Nitidulidae, new records, Canada, New Brunswick

## Abstract

We report 20 new species records for the Coleoptera fauna in New Brunswick, Canada, five of which are new records for the Maritime provinces, including one species that is new for Canada. One species of Kateretidae, *Kateretes pusillus* (Thunberg) is newly recorded for New Brunswick and the Maritime provinces. *Stelidota octomaculata* (Say), *Phenolia grossa* (Fabricius), and*Cryptarcha strigatula* Parsons of the family Nitidulidae are added to the faunal list of New Brunswick; the latter species is new to the Maritime provinces. Two species of Cerylonidae, *Philothermus glabriculus* LeConte and *Cerylon unicolor* (Ziegler), are reported for the first time for New Brunswick. *Philothermus glabriculus* is new for the Maritime provinces. Two species of Endomychidae, *Hadromychus chandleri* Bousquet and Leschen and *Danae testacea* (Ziegler) are newly recorded for New Brunswick. Three species of Coccinelidae, *Stethorus punctum punctum* (LeConte), *Naemia seriata seriata* Melsheimer, and *Macronaemia episcopalis* (Kirby) are added to the provincial list. *Macronaemia episcopalis* (Kirby) is a species new to the Maritime provinces. Nine species of Latridiidae, *Cartodere nodifer* (Westwood), *Dienerella ruficollis* (Marsham), *Enicmus aterrimus* Motschulsky, *Enicmus fictus* Fall, *Encimus histrio* Jay and Tomlin, *Lathridius minutus* (Linnaeus), *Stephostethus productus* Rosenhauer, *Corticaria elongata* (Gyllenhal), and *Corticarina longipennis* (LeConte) are newly recorded for New Brunswick. *Stephostehus productus* is newly recorded from Canada. Collection and habitat data are presented for all these species.

## Introduction

This paper treats new records from New Brunswick, Canada of the Coleoptera families Kateretidae, Nitidulidae, Cerylonidae, Endomychidae, Coccinellidae, and Latridiidae. The fauna of these families from New Brunswick and the Maritime provinces (New Brunswick, Nova Scotia, and Prince Edward Island) was recently treated by Majka and McCorquodale (2006), Majka and Robinson (2009) (Coccinellidae), Majka (2007), Majka (2009) (Endomychidae), Majka et al. (2008) (Kateretidae and Nitidulidae), Majka et al. (2009) (Latridiidae), and Majka and Langor (2011) (Cerylonidae). Intensive sampling in New Brunswick by the first author since 2003 and records obtained from a study to develop a general attractant for the detection of invasive Cerambycidae species have yielded additional new provincial records in the above families. This paper reports on these new records and provides a brief synopsis of each family.

## Methods and conventions

The following records are based on specimens collected during a general survey by the first author to document the Coleoptera fauna of New Brunswick and from by-catch samples obtained during a study to develop a general attractant for the detection of invasive species of Cerambycidae. Additional records were obtained from specimens contained in the collection of the Natural Resources Canada, Canadian Forest Service - Atlantic Forestry Centre, Fredericton, New Brunswick.

### Collection methods

Various methods were employed to collect the specimens and details are outlined in Webster et al. (2009, Appendix). Many specimens were also collected from 12-unit Lindgren funnel traps set in various forest habitats in New Brunswick between 2008 and 2011. These traps visually mimic tree trunks and are often effective for sampling species of Coleoptera that live in microhabitats associated with standing trees (Lindgren 1983). See Webster et al. (in press) for details of the methods used to deploy Lindgren 12-funnel traps and for sample collection. A description of the habitat was recorded for all specimens collected during this survey. Locality and habitat data are presented exactly as on labels for each record. This information, as well as additional collecting notes, is summarized and discussed in the collection and habitat data section for each species.

### Distribution

Distribution maps, created using ArcMap and ArcGIS, are presented for each species in New Brunswick. Every species is cited with its currently known distribution in Canada and Alaska, using abbreviations for the state, provinces, and territories. New records for New Brunswick are indicated in bold under Distribution in Canada and Alaska. The following abbreviations are used in the text:

**Table:** 

**AK**	Alaska	**MB**	Manitoba
**YT**	Yukon Territory	**ON**	Ontario
**NT**	Northwest Territories	**QC**	Quebec
**NU**	Nunavut	**NB**	New Brunswick
**BC**	British Columbia	**PE**	Prince Edward Island
**AB**	Alberta	**NS**	Nova Scotia
**SK**	Saskatchewan	**NF & LB**	Newfoundland and Labrador*

*Newfoundland and Labrador are each treated separately under the current Distribution in Canada and Alaska.

Acronyms of collections examined or where voucher specimens reside are as follows:

**AFC** Atlantic Forestry Centre, Natural Resources Canada, Canadian Forest Service, Fredericton, New Brunswick, Canada

**CNC** Canadian National Collection of Insects, Arachnids and Nematodes, Agriculture and Agri-Food Canada, Ottawa, Ontario, Canada

**MTC** Martin Turgeon Collection, Saint Basil, New Brunswick, Canada

**NBM** New Brunswick Museum, Saint John, New Brunswick, Canada

**RWC** Reginald P. Webster Collection, Charters Settlement, New Brunswick, Canada

## Results

### Species accounts

All records below are species newly recorded for New Brunswick, Canada. Species followed by ** are newly recorded from the Maritime provinces of Canada. Species followed by *** are newly recorded for Canada.

### Family Kateretidae Kirby, 1837

The Kateretidae (the short-winged flower beetles) are phytophagous both as larvae and adults (Habeck 2002a). Larvae develop in seed capsules, and adults feed on flower petals and pollen. The Kateretidae (including Nitidulidae) of New Brunswick was reviewed by Majka et al. (2008). Four species were recorded for the province, including *Brachypterus urticae* (Fabricius*), Heterhelus abdominalis* (Erichson), and *Heterhelus sericans* (LeConte), which were newly reported for New Brunswick. Here, we newly record *Kateretes pusillus* (Thunberg) for New Brunswick and the Maritime provinces (see Table 1).

**Table 1.** Species of Kateretidae, Cerylonidae, Endomychidae, and Latridiidae known from New Brunswick, Canada.

**Kateretidae Kirby**
*Brachypterolus pulicarius* (Linnaeus)
*Brachypterus urticae* (Fabricius)
*Heterhelus abdominalis* (Erichson)
*Heterhelus sericans* (LeConte)
*Kateretes pusillus* (Thunberg)**
**Family Cerylonidae Billberg**
**Subfamily Ceryloninae Billberg**
*Cerylon castaneum* Say
*Cerylon unicolor* (Ziegler)*
*Philothermus glabriculus* LeConte**
**Family Endomychidae Leach**
**Subfamily Endomychinae Leach**
*Endomychus biguttatus* Say
**Subfamily Epipocinae Gorham**
*Hadromychus chandleri* Bousquet & Leschen*
**Subfamily Leiestinae Thomson**
*Phymaphora pulchella* Newman
**Subfamily Lycoperdininae Bromhead**
*Lycoperdina ferruginea* LeConte
*Mycetina perpulchra* (Newman)
**Subfamily Stenotarsinae Chapuis**
*Danae testacea* (Ziegler)*
**Family Latridiidae Erichson**
**Subfamily Latridiinae Erichson**
*Cartodere (Cartodere) constrcta* (Gyllenhal)
*Cartodere (Aridius) nodifer* (Westwood)*
*Dienerella argus* (Reitter)
*Dienerella ruficollis* (Marsham)*
*Enicmus aterrimus* Motschulsky*
*Enicmus fictus* Fall**
*Enicmus histrio* Joy & Tomlin*
*Enicmus tenuicornis* LeConte
*Lathridius consimilis* (Mannerheim)
*Lathridius minutus* (Linnaeus)*
*Stephostethus breviclavis* (Fall)
*Stephostethus litratus* (LeConte)
*Stephostethus productus* Rosenhauer***
*Thes bergrothi* (Reitter)
**Subfamily Cortcarinae Curtis**
*Corticaria elongata* (Gyllenhal)*
*Corticaria ferruginea* Marsham
*Corticaria impressa* (Olivier)
*Corticaria rubripes* Mannerheim
*Corticaria saginata* Mannerheim
*Corticarina cavicollis* (Mannerheim)
*Corticarina longipennis* (LeConte)*
*Corticarina minuta* (Fabricius)
*Cortinicara gibbosa* (Herbst)
*Melanophthalma helvola* Motschulsky
*Melanophthalma inermis* Motschulsky
*Melanophthalma picta* (LeConte)

**Notes:** *New to province, **New to Maritime provinces, *** New to Canada.

#### Kateretes pusillus

(Thunberg, 1794)**

http://species-id.net/wiki/Kateretes_pusillus

Map 1

##### Material examined.

**New Brunswick, Restigouche Co.**, Wild Goose Lake, 47.8539°N, 68.3219°W, 7.VI.2011, 20.VI.2011, R. Webster & M. Turgeon, lake margin, *Carex* marsh, treading *Carex* (21, AFC, MTC, NBM, RWC); Kedgwick Road at Fog Brook, 47.8367°N, 67.8739°W, 21.VI.2011, R. P. Webster, *Carex* marsh near brook, treading *Carex* (2, NBM, RWC).

**Map 1.**
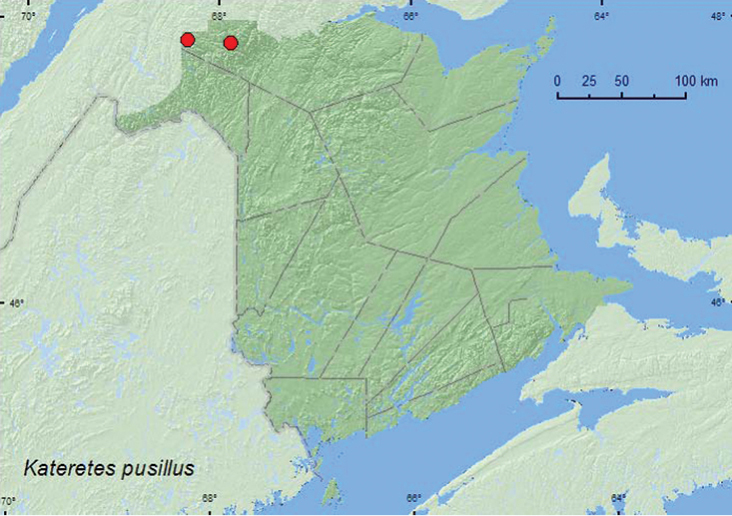
Collection localities in New Brunswick, Canada of *Kateretes pusillus*.

##### Collection and habitat data.

Adults of this northern species were collected by treading (forcing emergent vegetation into water) *Carex* in *Carex* marshes along a lake margin and a brook. At both sites where this species was found, *Carex* was covered with pollen. Adults were captured during June. Specimens of this species in the Carr collection in the CNC were collected by sweeping or sieving sedges in beaver (*Castor canadensis* Kuhl.) ponds, marshes, bogs, dried boggy areas, small muskegs, sedge marshes, swamps, in sphagnum and moss at the edge of a sedge marsh, in wash-up along a river, and by sifting willow (*Salix*)/poplar (*Populus*) leaves on a slope around a marsh (Anthony Davies, personal communication).

##### Distribution in Canada and Alaska.

AK, NT, AB, SK, ON, QC, **NB** (McNamara 1991b).

### Family Nitidulidae Latreille, 1802

The Nitidulidae (the sap beetles) is a large family of mostly saprophagous and mycetophagous species (Habeck 2002b) with many taxa found in decaying fruit, in fermenting plant juices and sap, on fungal sporocarps, and others on flowers. *Nitidula* sp. and *Omosita* sp. are found in carrion, and a few species are minor stored-product pests (Habeck 2002b). The Nitidulidae (as well as Kateretidae) of New Brunswick was reviewed by Majka et al. (2008). Forty-two species were recorded for the province, 28 were newly reported. Here, we report three additional species from the province. See Majka et al. (2008) for a list of the other Nitidulidae species known from New Brunswick.

### Subfamily Nitidulinae Latreille, 1802

#### Stelidota octomaculata

(Say, 1825)

http://species-id.net/wiki/Stelidota_octomaculata

Map 2

##### Material examined.

**New Brunswick, Queens Co.**, Cranberry Lake P.N.A (Protected Natural Area), 46.1125°N, 65.6075°W, 2.IX.2009, R. Webster & M.-A. Giguère, old red oak forest, in nest of black *Formica* species (mound building species) (1, RWC); same locality data and forest type, 31.VIII–15.IX.2011, C. Hughes & R. P. Webster, Lindgren funnel traps (2, RWC).

**Map 2.**
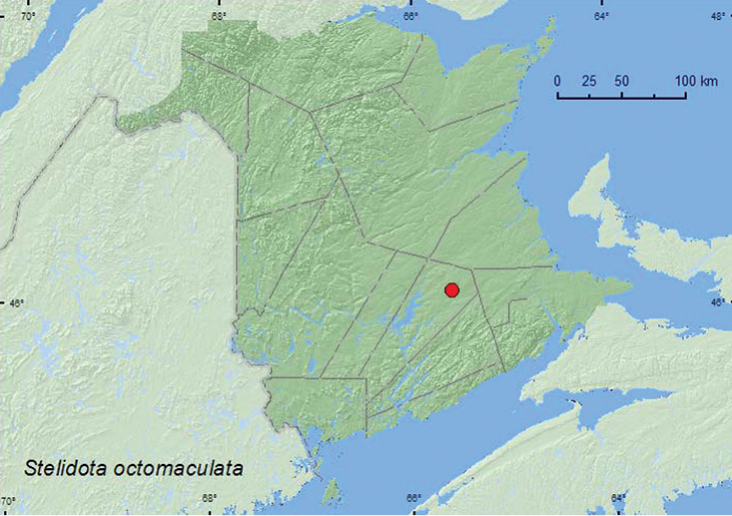
Collection localities in New Brunswick, Canada of *Stelidota octomaculata*.

##### Collection and habitat data.

*Stelidota octomaculata* has been reported from decaying fruit and fungi, coral fungi (Downie and Arnett 1996), gilled mushrooms, decaying pigs, and from pitfall traps deployed in a hardwood forest (Majka and Cline 2006). One specimen from New Brunswick was found in a *Formica* ant nest in an old red oak (*Quercus rubra* L.) stand; two others were captured in Lindgren funnel traps in the same forest. Adults were collected during September.

##### Distribution in Canada and Alaska.

ON, QC, **NB**, NS (McNamara 1991b; Majka and Cline 2006).

#### Phenolia grossa

(Fabricius, 1801)

http://species-id.net/wiki/Phenolia_grossa

Map 3

##### Material examined.

**New Brunswick, Carleton Co.**, Meduxnekeag Valley Nature Preserve, 46.1940°N, 67.6801°W, 12.IX.2008, R. P. Webster, mixed forest, in *Laetiporus sulphureus* (3, RWC); same locality but 46.1887°N, 67.6735°W, 13.VI.2010, R. P. Webster, hardwood forest, in *Laetiporus sulphureus* (8, NBM, RWC). **Queens Co.**, Cranberry Lake P.N.A, 46.1125°N, 65.6075°W, 7.VI-22.VI.2011, M. Roy & V. Webster, old red oak forest, Lindgren funnel trap (1, NBM); same locality data and forest type, 4.VIII.2011, 18.VIII.2011, R. P. Webster, in *Laetiporus sulphureus* (5, AFC, NBM, RWC).

**Map 3.**
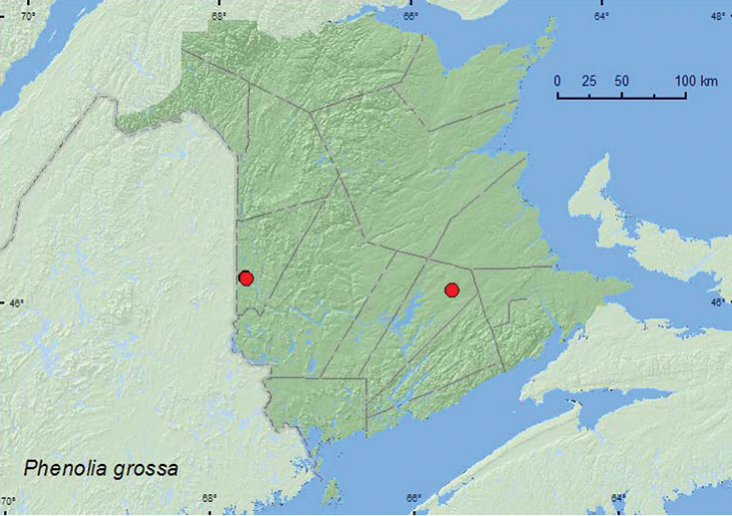
Collection localities in New Brunswick, Canada of *Phenolia grossa*.

##### Collection and habitat data.

Majka and Cline (2006) reported this species mostly from coniferous forests in Nova Scotia and from one hardwood stand. Adults were reported from decaying red maple (*Acer rubrum* L.) and decomposing fungi. In New Brunswick, adults were collected from *Laetiporus sulphureus* (Fr.) Murr. (chicken mushroom) in a hardwood forest with sugar maple (*Acer saccharum* Marsh.), white ash (*Fraxinus americana* L.), and American beech (*Fagus grandifolia* Ehrh.) and in an old red oak forest. One individual was captured in a Lindgren funnel trap deployed in an old red oak stand. Adults were collected during June, August, and September.

##### Distribution in Canada and Alaska.

ON, QC, **NB**, NS (McNamara 1991b; Majka and Cline 2006).

### Subfamily Cryptarchinae Thomson, 1859

**Tribe Cryptarchini Thomson, 1859**

#### Cryptarcha strigatula

Parsons, 1938**

http://species-id.net/wiki/Cryptarcha_strigatula

Map 4

##### Material examined.

**New Brunswick, Queens Co.**, Cranberry Lake P.N.A, 46.1125°N, 65.6075°W, 7-22.VI.2011, M. Roy & V. Webster, mature red oak forest, Lindgren funnel traps (2, RWC). Charters Settlement, 45.8395°N, 66.7391°W, 20.VII.2006, 1.VIII.2007, R. P. Webster, mixed forest, m.v. light (2, RWC).

**Map 4.**
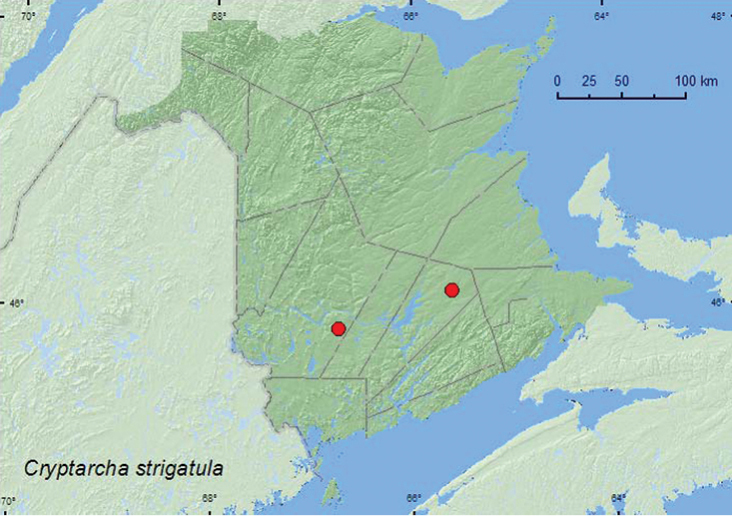
Collection localities in New Brunswick, Canada of *Cryptarcha strigatula*.

##### Collection and habitat data.

This species was captured in Lindgren funnel traps deployed in an old red oak forest and at a mercury-vapor light near a mixed forest. Adults were collected during June, July, and August.

##### Distribution in Canada and Alaska.

ON, QC, **NB.** There are two specimens in the CNC from Canada with the following data: Ont: Leeds County, Leeds and Lansdowne Township, 17.VIII.1992, *Quercus alba* under bark; Que: Co. Iberville, Rivière du Sud, 30.VII.1975, N. Doiron, CH308 (Anthony Davies, personal communication).

### Family Cerylonidae Billberg, 1820

North American species of Cerylonidae (the minute bark beetles) were revised by Lawrence and Stephan (1975). Nineteen species were recorded for North America (Lawrence and Stephan 1975), seven from Canada, and only *Cerylon castaneum* Say from New Brunswick (Campbell 1991a). Majka and Langor (2011) reviewed the Cerylonidae of Atlantic Canada but did not report any additional species for New Brunswick. Adults are found under bark, in leaf litter, or in rotten wood and probably feed on fungi (Campbell 1991b; Thomas 2002). The adventive *Murmidius ovalis* (Beck) occurs in stored products (Lawrence and Stephan 1975). Here, we report two species of Cerylonidae that are new for New Brunswick (Table 1). *Philothermus glabriculus* LeConte is newly recorded for the Maritime provinces.

### Ceryloninae Billberg, 1820

#### Philothermus glabriculus

LeConte, 1863**

http://species-id.net/wiki/Philothermus_glabriculus

Map 5

##### Material examined.

**New Brunswick, Carleton Co.**, Jackson Falls, Bell Forest, 46.2200°N, 67.7231°W, 4-12.VI.2008, R. P. Webster, mature hardwood forest, Lindgren funnel traps (2, AFC, NBM). **Queens Co.**, Cranberry Lake P.N.A., 46.1125°N, 65.6075°W, 25.VI-1.VII.2009, R. Webster & M.-A. Giguère, mature red oak forest, Lindgren funnel trap (1, RWC); same locality and habitat data, 7-22.VI.2011, 29.VI-7.VII.2011, 7-13.VII.2011, M. Roy & V. Webster, Lindgren funnel traps (7, AFC, NBM, RWC). **Sunbury Co.**, Acadia Research Forest, 45.9866°N, 66.3841°W, 24-30.VI.2009, R. Webster & M.-A. Giguère, mature (110 year-old) red spruce forest with scattered red maple and balsam fir, Lindgren funnel trap (1, RWC). **York Co.**, 15 km W of Tracy off Rt. 645, 45.6848°N, 66.8821°W, 7-14.VII.2010, R. Webster & C. MacKay, old red pine forest, Lindgren funnel trap (1, RWC); 14 km WSW of Tracy, S of Rt. 645, 45.6741°N, 66.8661°W, 16-30.VI.2010, R. Webster & C. MacKay, old mixed forest with red and white spruce, red and white pine, balsam fir, eastern white cedar, red maple, and *Populus* sp., Lindgren funnel trap (1, AFC).

**Map 5.**
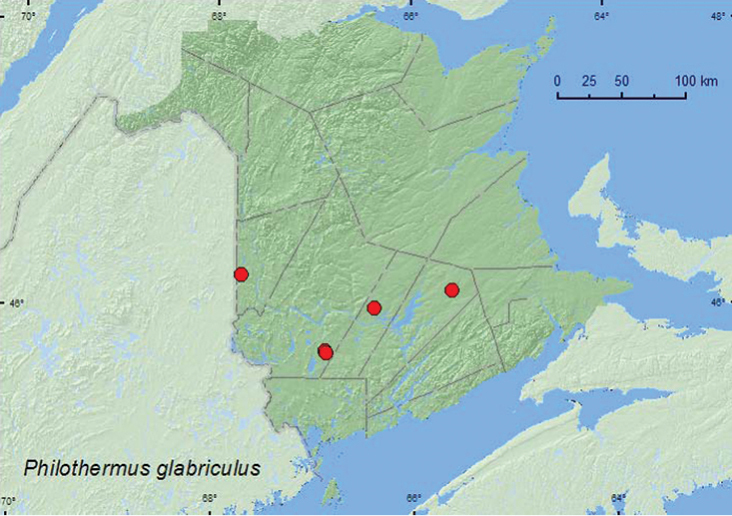
Collection localities in New Brunswick, Canada of *Philothermus glabriculus*.

##### Collection and habitat data.

*Philothermus glabriculus* was collected in various forest types in New Brunswick, including a mature hardwood forest with American beech, sugar maple, white ash, a mature red oak forest, a mature red spruce (*Picea rubens* Sarg.) forest, an old red pine (*Pinus resinosa* Ait.) forest, and an old mixed forest. Adults were captured in Lindgren funnel traps during June and July.

##### Distribution in Canada and Alaska.

ON, QC, **NB** (Campbell 1991a).

#### Cerylon unicolor

(Ziegler, 1845)

http://species-id.net/wiki/Cerylon_unicolor

Map 6

##### Material examined.

**New Brunswick, Carleton Co.**, Jackson Falls, Bell Forest, 46.2200°N, 67.7231°W, 6.V.2007, R. P. Webster, mature hardwood forest, on fleshy polypore (bracket) fungi on dead standing beech (1, RWC); same locality and forest type but 1-8.VI.2009, 16-21.VI.2009, 21-28.VI.2009, 7-14.VII.2009, R. Webster & M.-A. Giguère, Lindgren funnel traps (4, AFC, RWC); Meduxnekeag Valley Nature Preserve, 46.1907°N, 67.6740°W, 7.VI.2007, R. P. Webster, hardwood forest, under bark of sugar maple log (1, RWC). **Charlotte Co.**, 10 km NW of New River Beach, 45.2110°N, 66.6170°W, 15-29.VI.2010, R. Webster & C. MacKay, old growth eastern white cedar forest, Lindgren funnel trap (1, AFC). **Queens Co.**, Cranberry Lake P.N.A., 46.1125°N, 65.6075°W, 5-11.VI.2009, 25.VI-1.VII.2009, R. Webster & M.-A. Giguère, mature red oak forest, Lindgren funnel trap (2, NBM, RWC). **Restigouche Co.**, Kedgwick Forks, 47.9085°N, 67.9057°W, 22.VI.2010, river margin, in flood debris (1, NBM). **Sunbury Co.**, Portobello Creek N.W.A., Maugerville, 45.8990°N, 66.4200°W, 28.VI.2004, R. P. Webster, silver maple swamp, under bark of log (1, RWC); Acadia Research Forest, 45.9866°N, 66.3841°W, 16-24.VI.2009, 24-30.VI.2009, R. Webster & M.-A. Giguère, mature (110 year-old) red spruce forest with scattered red maple and balsam fir, Lindgren funnel traps (5, AFC). **York Co.**, Charters Settlement, 45.8188°N, 66.7460°W, 25.VIII.2004, R. P. Webster, clear-cut, under bark of conifer stump (3, RWC); same locality but 45.8286°N, 66.7365°W, 2.VI.2007, R. P. Webster, mature red spruce forest, under bark of red spruce (1, RWC); 15 km W of Tracy off Rt. 645, 45.6848°N, 66.8821°W, 15-21.VI.2009, R. Webster & M.-A. Giguère, old red pine forest, Lindgren funnel traps (2, AFC); 14 km WSW of Tracy, S of Rt. 645, 45.6741°N, 66.8661°W, 10-26.V.2010, R. Webster & C. MacKay, old mixed forest with red and white spruce, red and white pine, balsam fir, eastern white cedar, red maple, and *Populus* sp., Lindgren funnel trap (1, AFC).

**Map 6.**
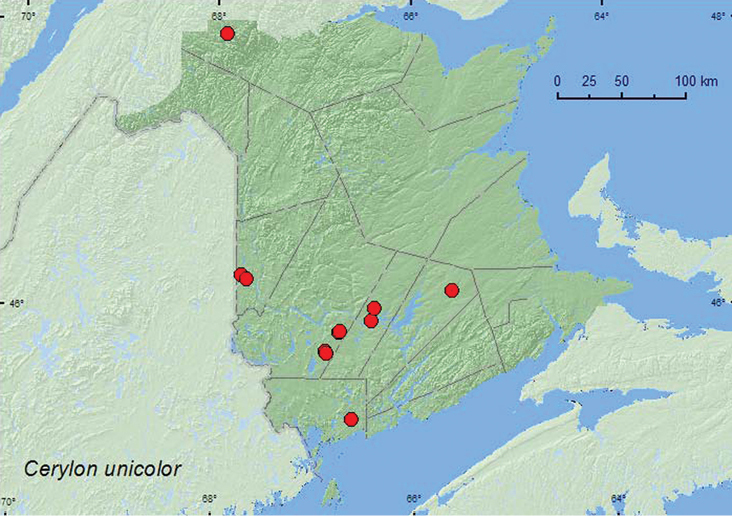
Collection localities in New Brunswick, Canada of *Cerylon unicolor*.

##### Collection and habitat data.

In Nova Scotia, this species was found in red spruce stands on *Fomitopsis officinalis* (Fr.) Bond. & Sing., in an old-growth hemlock (*Tsuga canadensis* (L.) Carr.) forest, and in a mixed old-growth hemlock, black spruce (*Picea mariana* (Mill.) B.S.P.), and balsam fir (*Abies balsamea* (L.) Mill.) stand (Majka and Langor 2011). This species has been reported from under bark of various hardwood and conifer species, and on fungi, such as *Bjerkandera adusta* (Fr.) Kar. and *Phellinus gilvus* (Schw.) Pat. (Lawrence and Stephan 1975). In New Brunswick,*Cerylon unicolor* was collected from under bark of sugar maple, silver maple (*Acer saccharinum* L.), red spruce, and a conifer stump. One individual was sifted from flood debris along a river margin, another was found in fleshy polypore fungi on a dead, standing American beech tree. This species was also captured in Lindgren funnel traps deployed in hardwood forests with sugar maple and American beech, mixed forests, a mature red oak forest, an old red pine forest, a mature red spruce forest, and an old eastern white cedar (*Thuja occidentalis* L.) forest. Adults were captured during May, June, and July.

##### Distribution in Canada and Alaska.

NT, BC, AB, ON, QC, **NB**, NS, NF (Campbell 1991a; Majka and Langor 2011).

### Family Endomychidae Leach, 1815

The Endomychidae (handsome fungus beetles) are found in subcortical fungi, soft polypores, fleshy basidiomycetes, and various molds and mildews or are specialists on puffballs (*Lycoperdina ferruginea* LeConte) (Skelley and Leschen 2002). The Endomychidae (and Erotylidae) of the Maritime provinces were reviewed by Majka (2007). *Phymatphora pulchella* Newman and *Rhanidea unicolor* (Ziegler) (Endomychidae) were reported from the province for the first time. However, the determination of *Rhanidea unicolor* was in error and was a specimen of *Lycoperdina ferruginea* LeConte, a species new to New Brunswick (Majka 2009). *Rhanidea unicolor* was, therefore, removed from the faunal list of the province. Majka (2007) discussed the fungal associations of members of this family from the Maritime provinces and the impact that forest management practices may have on the communities of forest fungi and the associated beetle species dependent on these fungi. Four species of Endomychidae were reported from New Brunswick by Majka (2007, 2009). Here, we add two species to the faunal list of New Brunswick (Table 1).

### Subfamily Epipocinae Gorham, 1873

#### Hadromychus chandleri

Bousquet & Leschen, 2002

http://species-id.net/wiki/Hadromychus_chandleri

Map 7

##### Material examined.

**New Brunswick, Carleton Co.**, Jackson Falls, Bell Forest, 46.2200°N, 67.7231°W, 4-12.VI.2008, 12-19.VI.2008, R. P. Webster, mature hardwood forest, Lindgren funnel traps (5, NBM, RWC); same locality and habitat data, 28.IV-9.V.2009, 9-14.V.2009, 14-20.V.2009, 21-28.VI.2009, R. Webster & M.-A. Giguère, mature hardwood forest, Lindgren funnel traps (8, AFC, RWC). **Queens Co.**, Cranberry Lake P.N.A., 46.1125°N, 65.6075°W, 12-21.V.2009, 21-27.V.2009, 27.V-5.VI.2009, R. Webster & M.-A. Giguère, old red oak forest, Lindgren funnel traps (5, AFC); same locality data and forest type, 25.V-7.VI.2011, M. Roy & V. Webster, Lindgren funnel trap (1, NBM). **Restigouche, Co.**, Dionne Brook P.N.A., 47.9030°N, 68.3503°W, 31.V-15.VI.2011, M. Roy & V. Webster, old-growth northern hardwood forest, Lindgren funnel traps (3, AFC, NBM); same locality and collectors but 47.9064°N, 68.3441°W, 31.V-15.VI.2011, 27.VI-14.VII.2011, old-growth white spruce and balsam fir forest (26, AFC, NBM, RWC). **Sunbury Co.**, Acadia Research Forest, 45.9866°N, 66.3841°W, 28.IV-8.V.2009, 13-19.V.2009, 19-25.V.2009, 2-9.VI.2009, 24-30.VI.2009, R. Webster & M.-A. Giguère, mature (110 year-old) red spruce forest with scattered red maple and balsam fir, Lindgren funnel traps (6, AFC, RWC). **York Co.**, 15 km W of Tracy off Rt. 645, 45.6848°N, 66.8821°W, 4-11.V.2009, 11-19.V.2009, R. Webster & M.-A. Giguère, old red pine forest, Lindgren funnel traps (2, AFC, RWC); 14 km WSW of Tracy, S of Rt. 645, 45.6741°N, 66.8661°W, 26.IV-10.V.2010, 10-26.V.2010, 26.V-2.VI.2010, R. Webster & C. MacKay, old mixed forest with red and white spruce, red and white pine, balsam fir, eastern white cedar, red maple, and *Populus* sp., Lindgren funnel traps (8, AFC);Charters Settlement, 45.8395°N, 66.7391°W, 1–5.VI.2011, R. P. Webster, mixed forest, flight intercept trap (1, NBM).

**Map 8.**
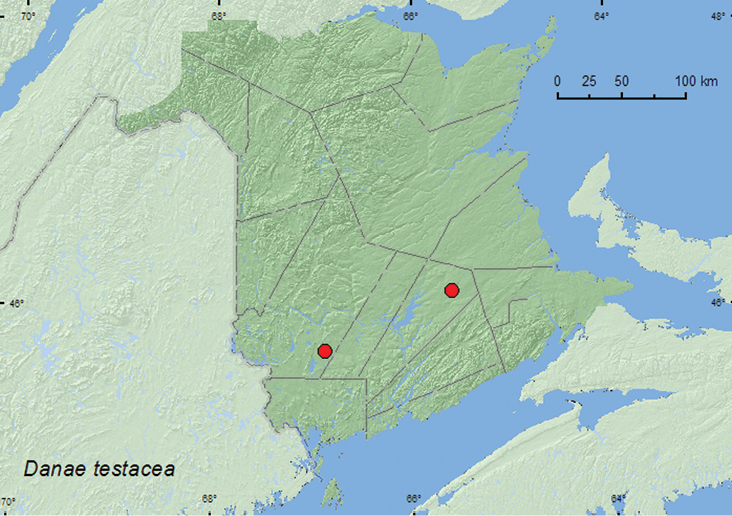
Collection localities in New Brunswick, Canada of *Danae testacea*.

##### Collection and habitat data.

Little is known about the habitat requirements of *Hadromychus chandleri*. All (64) specimens from New Brunswick were captured in Lindgren funnel traps, which visually mimic tree trunks and are often effective for sampling species of Coleoptera that live in microhabitats associated with standing trees (Lindgren 1983). This species may likely live in microhabitats associated with standing trees. Specimens of this specieswere collected from a various forest types in New Brunswick. Adults were collected in a mature hardwood forest, an old-growth northern hardwood forest with sugar maple and yellow birch (*Betula alleghaniensis* Britt.), an old red oak forest, a mature red spruce forest, an old red pine forest, an old-growth white spruce (*Picea glauca* (Moench) Voss) and balsam fir forest, and old mixed forests. Most adults were captured in an old-growth white spruce and balsam fir forest (boreal forest) in northwestern New Brunswick. This species is likely a northern and boreal faunal component. Adults were collected during April, May, June, and July.

##### Distribution in Canada and Alaska.

ON, QC, **NB**, NS (Bousquet and Leschen 2002). The type series of this species consisted of seven specimens originating from New Hampshire, Nova Scotia, Ontario, and Quebec (Bousquet and Leschen 2002). Majka (2007) reported five additional specimens from Nova Scotia. Majka (2007) suggested that *Hadromychus chandleri* may be the rarest North American endomychid species. However, 64 specimens of this species were collected in New Brunswick between 2008 and 2011, indicating that this species may be more common, at least locally, than previously thought. All specimens from New Brunswick were captured in Lindgren funnel traps, and those reported by Majka (2007) from Nova Scotia were caught in flight-intercept traps, further suggesting that more specialized sampling methods are required to document the occurrence of this species. This species was most abundant in an old-growth boreal forest with white spruce and balsam fir.

### Subfamily Stenotarsinae Chapius, 1876

#### Danae testacea

(Ziegler, 1844)

http://species-id.net/wiki/Danae_testacea

Map 8

##### Material examined.

**New Brunswick, Queens Co.**, Cranberry Lake P.N.A., 46.1125°N, 65.6075°W, 14-19.VIII.2009, R. Webster & M.-A. Giguère, old red oak forest, Lindgren funnel trap (1, RWC). **York Co.**, 15 km W of Tracy off Rt. 645, 45.6848°N, 66.8821°W, 29.VI-7.VIII.2009, R. Webster & M.-A. Giguère, old red pine forest, Lindgren funnel trap (1, RWC).

**Map 7.**
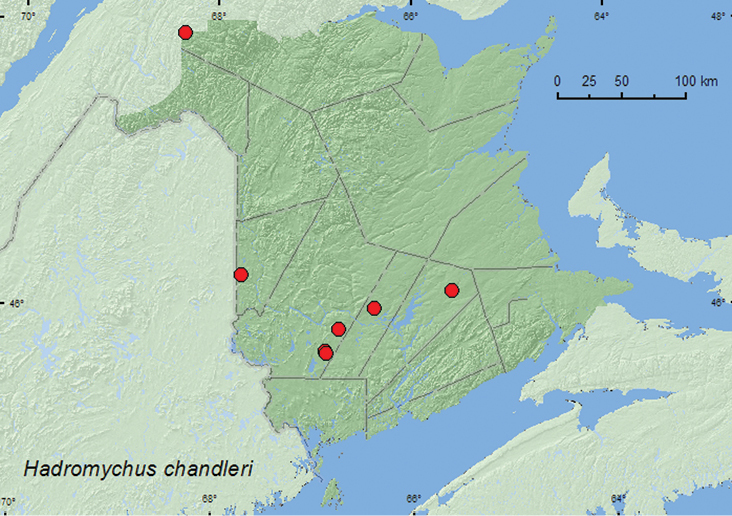
Collection localities in New Brunswick, Canada of *Hadromychus chandleri*.

##### Collection and habitat data.

*Danae testacea* was captured in Lindgren funnel traps in an old red oak forest and an old red pine forest. Both individuals of this species were captured during August. The specimen reported from Nova Scotia was found in an old-growth hardwood stand, and Majka (2007) suggested that this species may be associated with old-growth hardwood forests.

##### Distribution in Canada and Alaska.

ON, QC, **NB**, NS (Campbell 1991b).

### Family Coccinellidae Latreille, 1807

Majka and McCorquodale (2006) reviewed the Coccinellidae (the lady beetles) of the Maritime provinces. Later Majka and Robinson (2009) reviewed the *Hyperaspis* and *Brachiacantha* species in the Maritime provinces and provided keys to species. Thirty-nine species were reported from New Brunswick by Majka and McCorquodale (2006), but no new provincial records were reported. Three additional species are reported here, including *Macronaemia episcopalis* (Kirby), a species new to the Maritime provinces. See Majka and McCorquodale (2006) for a list of the other species of Coccinelidae known from New Brunswick.

### Subfamily Symninae

#### Stethorus punctum punctum

(LeConte, 1852)

http://species-id.net/wiki/Stethorus_punctum_punctum

Map 9

##### Material examined.

**New Brunswick, Charlotte Co.**, St. Andrews, 45.0751°N, 67.0374°W, 25.VIII.2009, R. P. Webster, sea beach, sweeping foliage (1, RWC). **Sunbury Co.**, Lakeville Corner, 45.9013°N, 66.2565°W, 27.VIII.2006, R. P. Webster, silver maple forest, on corncobs (1, RWC).

**Map 9.**
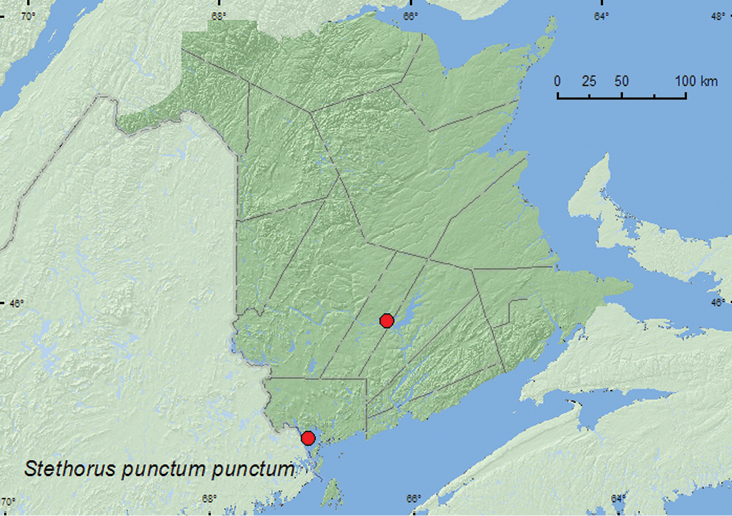
Collection localities in New Brunswick, Canada of *Stethorus punctatum punctatum*.

##### Collection and habitat data.

This species was collected by sweeping foliage on a sea beach and sifting debris from a pile of corncobs in a silver maple (*Acer saccharinum* L.) forest. The two adults were captured during August.

##### Distribution in Canada and Alaska.

AB, SK, ON, QC, **NB,** NS (McNamara 1991a).

### Subfamily Coccinellinae

#### Naemia seriata seriata

Melsheimer, 1847

http://species-id.net/wiki/Naemia_seriata_seriata

Map 10

##### Material examined.

**New Brunswick, Saint John Co.**, Dipper Harbour, 45.1169°N, 66.3771°W, 12.IX.2006, R. P. Webster, salt marsh, on flowers of seaside goldenrod (9 (many others observed), RWC); Chance Harbour off Cranberry Head Road, 45.1355°N, 66.3436°W, 30.V.2006, R. P. Webster, barrier beach, sweeping foliage of *Leucanthemum vulgare* Lam. (1, RWC); black beach, 45.1539°N, 66.2282°W, 11.VII.2008, R. P. Webster, sea beach, sweeping foliage (1, RWC).

**Map 10.**
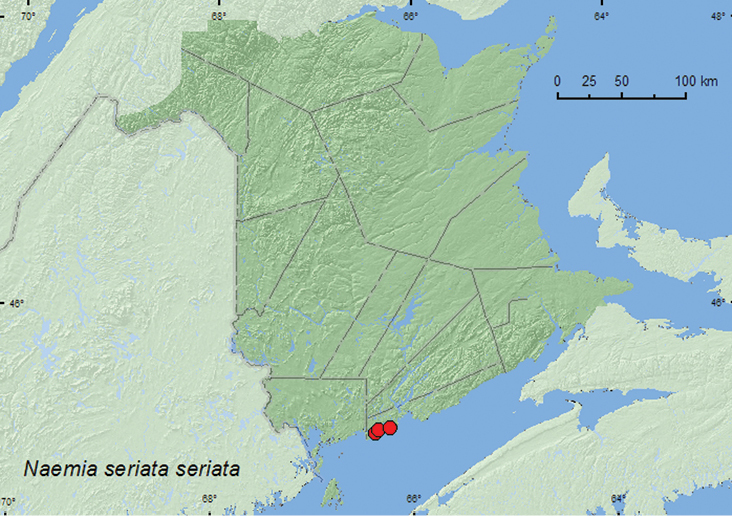
Collection localities in New Brunswick, Canada of *Naemia seriata seriata*.

##### Collection and habitat data.

Adults were taken by sweeping foliage of ox-eye daisy (*Leucanthemem vulgare* Lam.) on a barrier beach, sweeping foliage on a sea beach, and sweeping flowers of seaside goldenrod (*Solidago sempervirens* L.) in a salt marsh. Adults were taken during late May, July, and September.

##### Distribution in Canada and Alaska.

**NB,** NS (Majka and McCorquodale 2006). Majka and McCorquodale (2006) considered the Nova Scotia population as significantly disjunct from the remainder of its range (from southern Maine (Dearborn and Donahue 1993) to Central America (Gordon 1985)), and considered Nova Scotia at the northern limit of its environmental tolerances. This species is likely more widely distributed along the coast than originally thought and the distributional gaps may be the result of insufficient sampling in intervening areas.

#### Macronaemia episcopalis

(Kirby, 1837)**

http://species-id.net/wiki/Macronaemia_episcopalis

Map 11

##### Material examined.

**New Brunswick, Saint John Co.**, Dipper Harbour, 45.1169°N, 66.3771°W, 7.V.2006, R. P. Webster, margin of salt marsh, in debris on log (7, RWC); same locality, 12.IX.2006, R. P. Webster, salt marsh, sweeping vegetation (3, NBM, RWC).

**Map 11.**
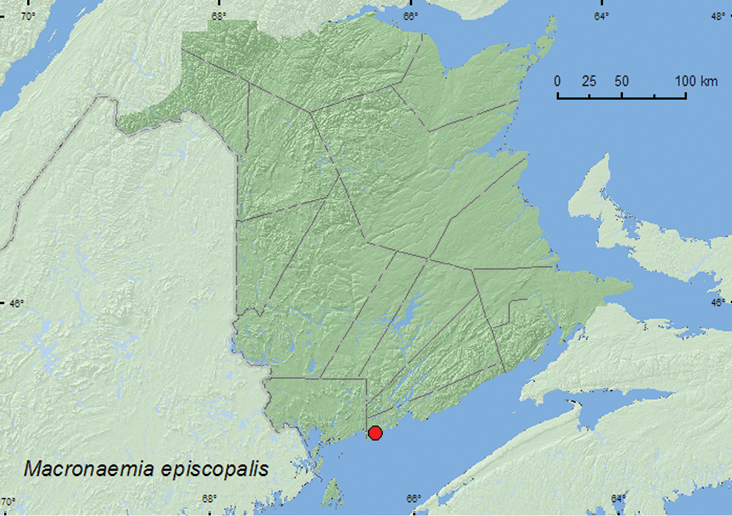
Collection localities in New Brunswick, Canada of *Macronaemia episcopalis*.

##### Collection and habitat data.

*Macronaemia episcopalis* was collected from salt marshes during September by sweeping foliage and sifting debris on a log in early May. The latter site was probably an overwintering site.

##### Distribution in Canada and Alaska.

AK, YK, NT, BC, AB, SK, MB, ON, QC, **NB** (McNamara 1991a). Gordon (1985) reported this species only as far east as western Quebec.

### Family Latridiidae Erichson, 1842

Andrews (2002) provided a general review of the Latridiidae (the minute brown scavenger beetles) of North America. Both adults and larvae feed on fungal conidia of Myxomycetes and can be found in leaf litter (Latridiinae) or by sweeping dead vegetation (Corticariinae). Some species occur in stored products (Andrews 2002). Bousquet (1991) reported 55 species for Canada and only six species for New Brunswick. Majka et al. (2009) reviewed the Latridiidae of the Atlantic Canada, provided keys to the known species from the region, and added 11 species to the faunal list of New Brunswick. Here, we report nine additional species for the province, including *Stephostehus productus* Rosenhauer, which is new for Canada.

### Subfamily Latridiinae Erichson, 1842

#### Cartodere (Aridius) nodifer

(Westwood, 1839)

http://species-id.net/wiki/Cartodere_nodifer

Map 12

##### Material examined.

**New Brunswick, Queens Co.**, Cranberry Lake P.N.A., 46.1125°N, 65.6075°W, 25.VI-1.VII.2009, 21-28.VII.2009, R. Webster & M.-A. Giguère, old red oak forest, Lindgren funnel traps (2, AFC, RWC). **York Co.**, Charters Settlement, 45.8395°N, 66.7391°W, 26.IX.2007, 30.IX.2007, 5.X.2007, 11.X.2007, 18.X.2007, R. P. Webster, mixed forest, in decaying (mouldy) corncobs and cornhusks (9, RWC).

**Map 12.**
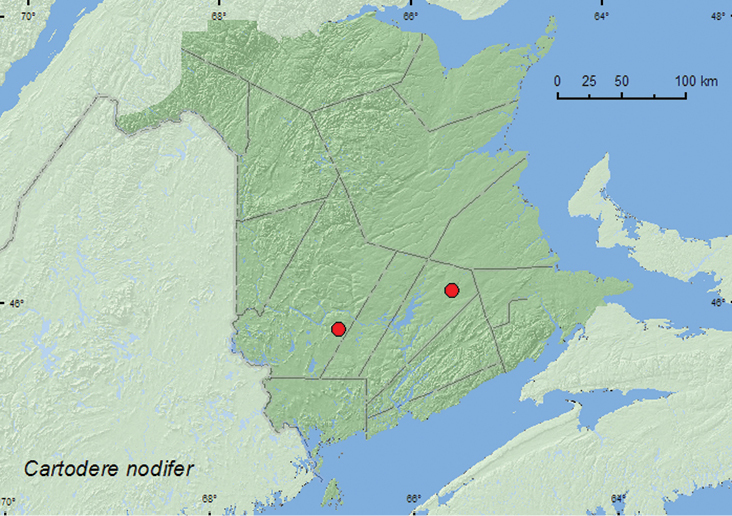
Collection localities in New Brunswick, Canada of *Cartodere nodifer*.

##### Collection and habitat data.

This adventive species is associated with stored products and has been found in various habitats promoting the growth of molds, such as under bark, in vegetable refuse, haystacks, and leaf compost (Hatch 1962; Bousquet 1990). Specimens from New Brunswick were sifted from decaying moldy corncobs and cornhusks, and captured in Lindgren funnel traps deployed in an old red oak forest. Adults were captured during June, July, September, and October.

##### Distribution in Canada and Alaska.

BC, MB, ON, QC, **NB**, NS, PE (Bousquet 1991; Majka et al. 2009).

#### Dienerella ruficollis

(Marsham, 1802)

http://species-id.net/wiki/Dienerella_ruficollis

Map 13

##### Material examined.

**New Brunswick, Kings Co.** Belle Isle (Bellisle Creek), II.18.1981 (no collector given), ex. bulk milk tank (20, AFC).

**Map 13.**
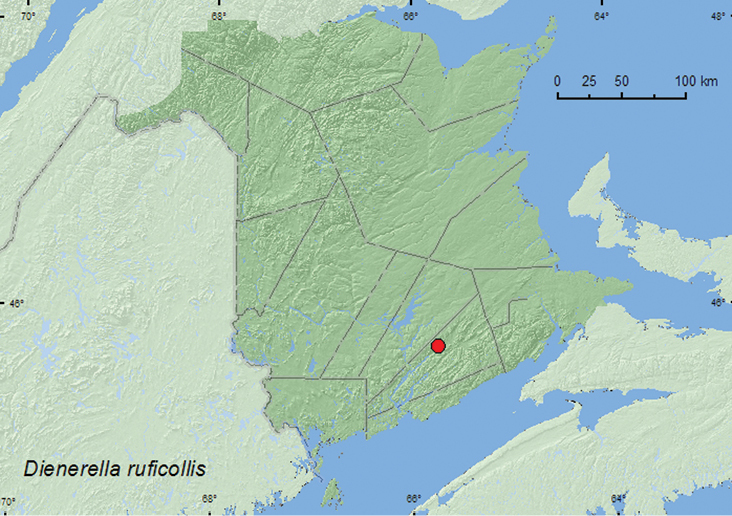
Collection localities in New Brunswick, Canada of *Dienerella ruficollis*.

##### Collection and habitat data.

A large series of this adventive Palaearctic species was collected from a bulk milk tank.

##### Distribution in Canada and Alaska.

BC, ON, QC, **NB**, NS, NF (Bousquet 1991).

#### Enicmus aterrimus

Motschulsky, 1866

http://species-id.net/wiki/Enicmus_aterrimus

Map 14

##### Material examined.

**New Brunswick, Carleton Co.**, Jackson Falls, Bell Forest, 46.2200°N, 67.7231°W, 5-15.VII.2008, R. P. Webster, mature hardwood forest, Lindgren funnel trap (1, NBM). **Queens Co.**, Cranberry Lake P.N.A., 46.1125°N, 65.6075°W, 24.IV-5.V.2009, 5-12.V.2009, 12-21.V.2009, R. Webster & M.-A. Giguère, old red oak forest, Lindgren funnel traps (17, AFC, NBM, RWC).

**Map 14.**
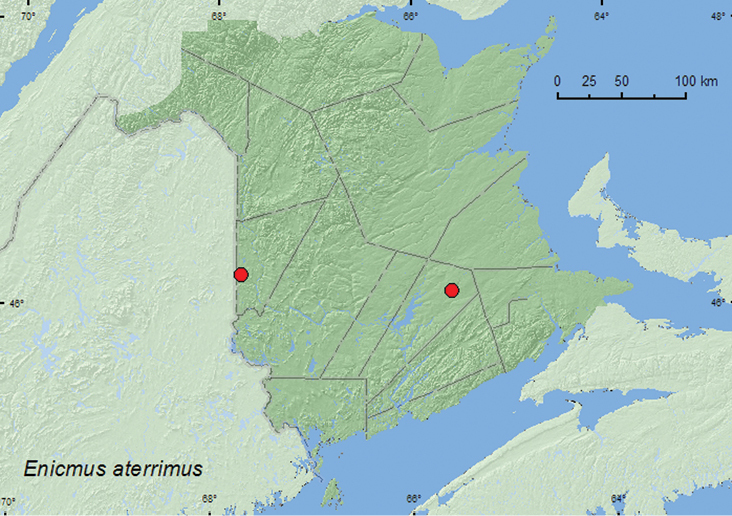
Collection localities in New Brunswick, Canada of *Enicmus aterrimus*.

##### Collection and habitat data.

This species was captured in Lindgren funnel traps deployed in an old red oak forest and a mature hardwood forest. Adults were captured during April, May, and July.

##### Distribution in Canada and Alaska.

ON, QC, **NB**, NS (Bousquet 1991; Majka et al. 2009). This species was first reported from Nova Scotia and Atlantic Canada by Majka et al. (2009).

#### Enicmus fictus

Fall, 1899**

http://species-id.net/wiki/Enicmus_fictus

Map 15

##### Material examined.

**New Brunswick, York Co.**, Charters Settlement, 45.8395°N, 66.7391°W, 27.VI.2007, R. P. Webster, mixed forest, u.v. light (1 AFC); same locality, habitat, and collector, 23.IV.2008, 9.V.2008, collected during aerial flight 15:00 to 18:00 h (2, RWC); same locality data and collector, 30.IX.2007, 11.X.2007, mixed forest, in decaying (mouldy) corncobs and cornhusks (2, RWC).

**Map 15.**
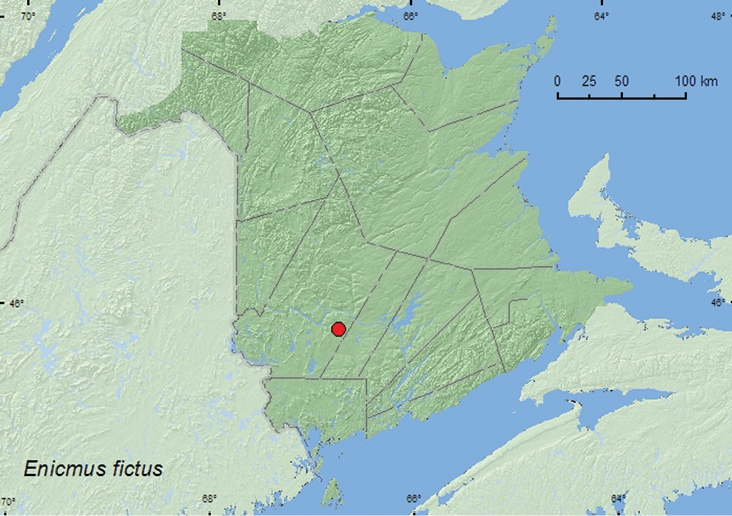
Collection localities in New Brunswick, Canada of *Enicmus fictus*.

##### Collection and habitat data.

This species is often found in stored products and has been collected from grass clippings (Hatch 1962; Bousquet 1990). In New Brunswick, specimens were collected from decaying (moldy) corncobs and cornhusks, at an ultraviolet light, and with an aerial net during an evening flight. Adults were captured during April, May, June, September, and October.

##### Distribution in Canada and Alaska.

AK, NT, BC, AB, SK, MB, ON, QC, **NB**, NF (Bousquet 1991; Majka et al. 2009).This species was newly recorded from Newfoundland and Atlantic Canada by Majka et al. (2009).

#### Encimus histrio

Joy and Tomlin, 1910

http://species-id.net/wiki/Encimus_histrio

Map 16

##### Material examined.

**New Brunswick, York Co.**, Marysville, 45.9750°N, 66.5700°W, 22.VI.2007, S. Makepeace & R. Webster, from nest material (remains of squirrel, various birds, bones, and insect parts) of barred owl in nest box (2, RWC).

**Map 16.**
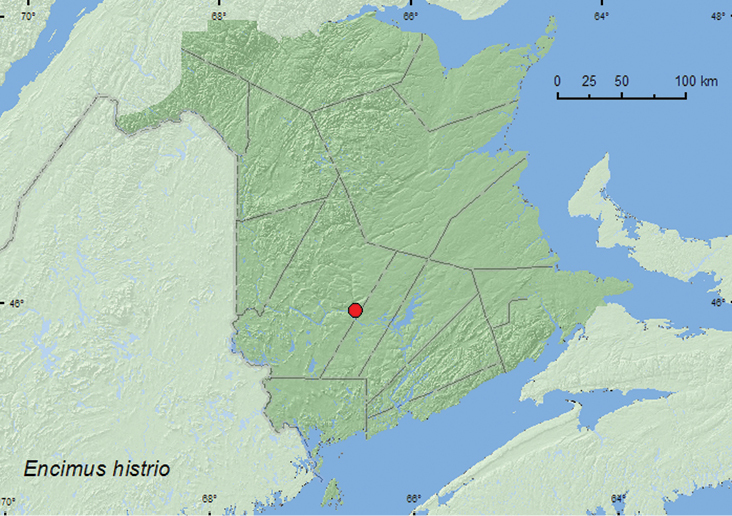
Collection localities in New Brunswick, Canada of *Enicmus histrio*.

##### Collection and habitat data.

Two individuals of this adventive Palaearctic species were collected from nest material from a barred owl (*Strix varia* Barton) nest box during June. In the Palaearctic, this species has been found in damp or moldy straw, hay, grass cuttings and vegetable refuse (Hinton 1945).

##### Distribution in Canada and Alaska.

**NB,** NS(Majka et al. 2009). This species was first reported from North America by Majka et al. (2009) from Sydney, Nova Scotia.

#### Lathridius minutus

(Linnaeus, 1767)

http://species-id.net/wiki/Lathridius_minutus

Map 17

##### Material examined.

**New Brunswick, Carleton Co.**, Jackson Falls, Bell Forest, 46.2200°N, 67.7231°W , 31.III.2005, R. P. Webster, mature hardwood forest, under bark of standing dead sugar maple (9, RWC); same locality and habitat data, 23-28.IV.2009, R. Webster & M.-A. Giguère, mature hardwood forest, Lindgren funnel traps (2, AFC, RWC). **Kings Co.**, Belle Isle (Bellisle Creek), 18.II.1981, (no collector given) from bulk milk tank (1, AFC).

**Map 17.**
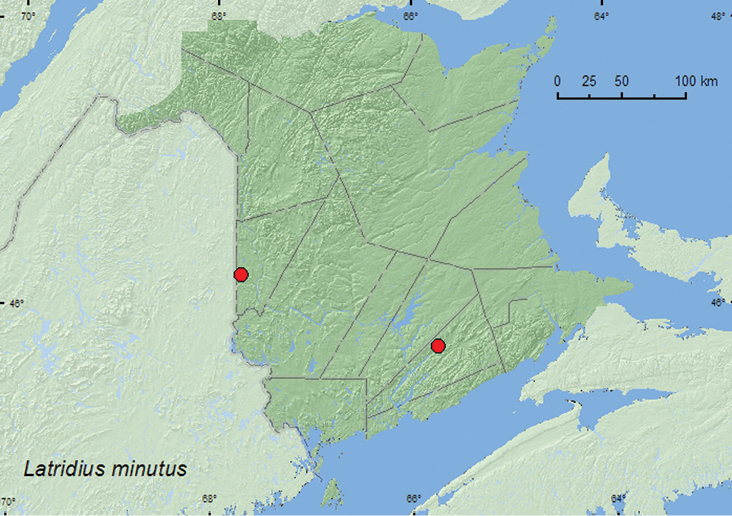
Collection localities in New Brunswick, Canada of *Lathridius minutus*.

##### Collection and habitat data.

In New Brunswick, thisadventive Palaearctic species was collected from under bark of a large, standing, dead sugar maple, from a bulk milk tank, and from Lindgren funnel traps deployed in a mature hardwood forest. Adults were captured during February, late March, and April. This species is commonly associated with stored products (Bousquet 1990). Majka et al. (2009) provide additional details on the habitat associations, bionomics, and timeline of the introduction of this species in North America.

##### Distribution in Canada and Alaska.

BC, AB, SK, MB, **NB**, PE, NS, NF (Bousquet 1991; Majka et al. 2009).

#### Stephostethus productus

Rosenhauer, 1856***

http://species-id.net/wiki/Stephostethus_productus

Map 18

##### Material examined.

**Canada, New Brunswick, York Co.**, 15 km W of Tracy off Rt. 645, 45.6848°N, 66.8821°W, 8-15.VI.2009, R. Webster & M.-A. Giguère, old red pine forest, Lindgren funnel trap (1, RWC).

**Map 18.**
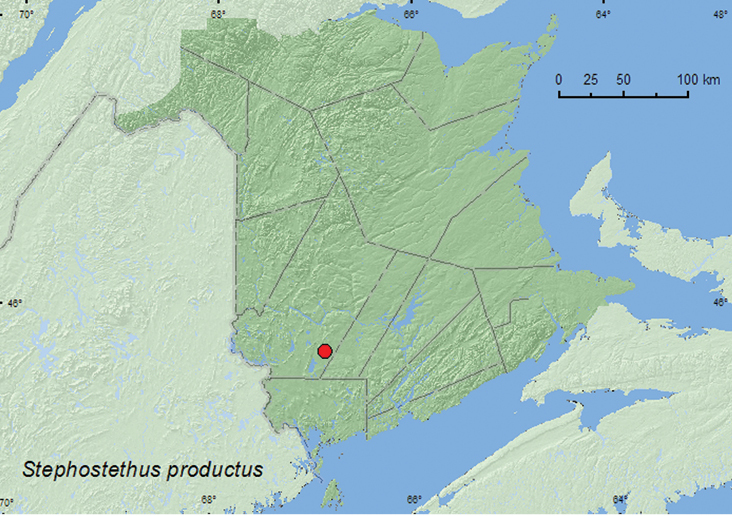
Collection localities in New Brunswick, Canada of *Stephostethus productus*.

##### Collection and habitat data.

One individual of this Palaearctic species was captured in a Lindgren funnel trap in an old red pine forest.

##### Distribution in Canada and Alaska.

**NB** **(new Canadian record).** Downie and Arnett (1996) reported this adventive Palaearctic species from the state of New York with a “?”, indicating that the record was questionable. We are not aware of any other records of this species for North America.

### Subfamily Corticariinae Curtis, 1829

#### Corticaria elongata

(Gyllenhal, 1827)

http://species-id.net/wiki/Corticaria_elongata

Map 19

##### Material examined.

**New Brunswick, Kings Co.**, Sussex, 18.IX.1981, (no collector given) from skim milk powder (2, AFC).

**Map 19.**
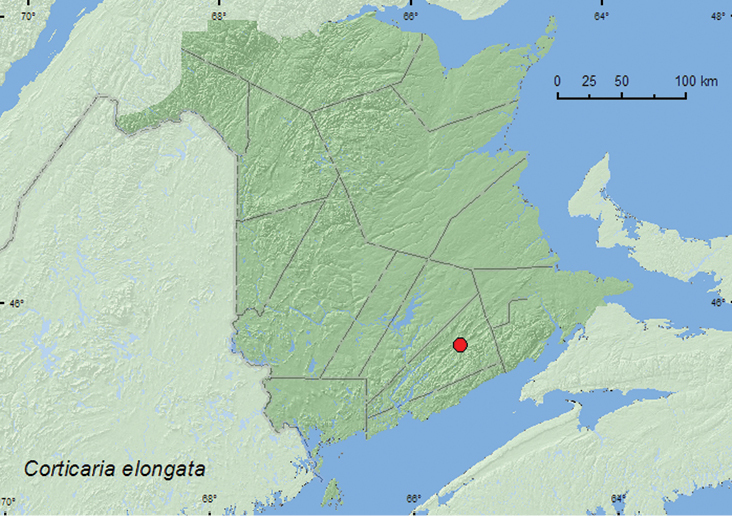
Collection localities in New Brunswick, Canada of *Corticaria elongata*.

##### Collection and habitat data.

Two individuals of this Holarctic or adventive Palaearctic species were collected from skim milk powder. In North America, this species is associated with stored products in grain elevators, warehouses, and feed mills (Hatch 1962; Bousquet 1990).

##### Distribution in Canada and Alaska.

**NB**, NS, NF (Majka et al. 2009). Majka et al. (2009) newly reported this for Canada on the basis of specimens from Newfoundland and Nova Scotia.

#### Corticarina longipennis

(LeConte, 1855)

http://species-id.net/wiki/Corticarina_longipennis

Map 20

##### Material examined.

**New Brunswick, Queens Co.**, Cranberry Lake P.N.A., 46.1125°N, 65.6075°W, 21-27.V.2009, R. Webster & M.-A. Giguère, old red oak forest, Lindgren funnel trap (1, RWC).

**Map 20.**
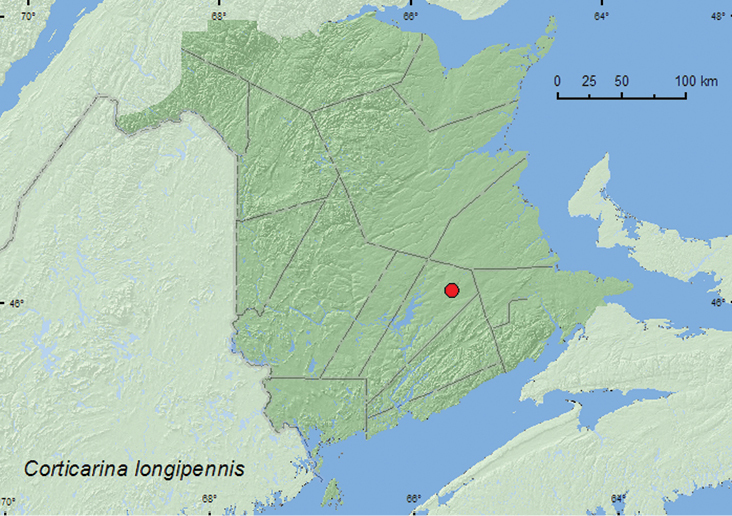
Collection localities in New Brunswick, Canada of *Corticarina longipennis*

##### Collection and habitat data.

One specimen was captured in a Lindgren funnel trap deployed in an old red oak forest. The adult was captured during May. Little is known about the habitat requirements of this species.

##### Distribution in Canada and Alaska.

**NB**, NS (Majka et al. 2009). Majka et al. (2009) reported this species for the first time for Canada from a specimen collected in Beaver River, Nova Scotia.

## Supplementary Material

XML Treatment for Kateretes pusillus

XML Treatment for Stelidota octomaculata

XML Treatment for Phenolia grossa

XML Treatment for Cryptarcha strigatula

XML Treatment for Philothermus glabriculus

XML Treatment for Cerylon unicolor

XML Treatment for Hadromychus chandleri

XML Treatment for Danae testacea

XML Treatment for Stethorus punctum punctum

XML Treatment for Naemia seriata seriata

XML Treatment for Macronaemia episcopalis

XML Treatment for Cartodere (Aridius) nodifer

XML Treatment for Dienerella ruficollis

XML Treatment for Enicmus aterrimus

XML Treatment for Enicmus fictus

XML Treatment for Encimus histrio

XML Treatment for Lathridius minutus

XML Treatment for Stephostethus productus

XML Treatment for Corticaria elongata

XML Treatment for Corticarina longipennis
